# Colorimetric Assay for Determination of Lead (II) Based on Its Incorporation into Gold Nanoparticles during Their Synthesis

**DOI:** 10.3390/s101211144

**Published:** 2010-12-07

**Authors:** Nan Ding, Qian Cao, Hong Zhao, Yimin Yang, Lixi Zeng, Yujian He, Kaixiang Xiang, Guangwei Wang

**Affiliations:** 1 College of Chemistry and Chemical Engineering, Graduate University of Chinese Academy of Sciences, 19A YuQuan Road, Beijing 100049, China; E-Mails: dingnaner@yahoo.com.cn (N.D.); cq56252581@yahoo.com.cn (Q.C.); yiminyang@gucas.ac.cn (Y.Y.); zenglixi79@126.com (L.Z.); 2 State Key Laboratory of Natural and Biomimetic Drugs, Peking University, Beijing 100191, China; 3 Huaihua Medical College, Hunan, 418000, China; E-Mail: hhyzggkb@163.com (K.X.); 4 Medical College, Hunan Normal University, Changsha, Hunan 410006, China

**Keywords:** visual detection, lead ions, gold nanoparticles, gallic acid

## Abstract

In this report, we present a new method for visual detection of Pb^2+^. Gold nanoparticles (Au-NPs) were synthesized in one step at room temperature, using gallic acid (GA) as reducer and stabilizer. Pb^2+^ is added during the gold nanoparticle formation. Analysis of Pb^2+^ is conducted by a dual strategy, namely, colorimetry and spectrometry. During Au-NPs synthesis, addition of Pb^2+^ would lead to formation of Pb-GA complex, which can induce the aggregation of newly-formed small unstable gold nanoclusters. Consequently, colorimetric detection of trace Pb^2+^ can be realized. As the Pb^2+^ concentration increases, the color turns from red-wine to purple, and finally blue. This method offers a sensitive linear correlation between the shift of the absorption band (Δλ) and logarithm of Pb^2+^ concentration ranging from 5.0 × 10^−8^ to 1.0 × 10^−6^ M with a linear fit coefficient of 0.998, and a high selectivity for Pb^2+^ detection with a low detection limit down to 2.5 × 10^−8^ M.

## 1. Introduction

Lead pollution is a serious danger to the environment and human health [1,2]. Intake of lead by human body can damage the kidneys, liver, and the gastrointestinal tract; besides, the nervous system and hemoglobin production will also be affected [3–7]. The maximum contamination level (MCL) for lead in drinking water is defined by the U.S. Environmental Protection Agency (EPA) to be 72 nM [8]. However, even at levels lower than 72 nM the presence of lead in children is associated with brain and neuro-developmental deficiencies [9–11]. Therefore, it is essential to develop sensors for ultrasensitive detection of Pb^2+^.

Several methods for Pb^2+^ analysis have been developed in the past decade, including ones based on atomic absorption spectrometry (AAS), atomic emission spectrometry (AES), inductively coupled plasma mass spectrometry (ICP-MS), anodic stripping voltammetry, and reversed-phase high-performance liquid chromatography coupled with UV-vis or fluorescence detection [12–17]. With regard to sensitivity and accuracy, these methods are all efficient tools for Pb^2+^ detection, but they are time-consuming, expensive, and/or require sophisticated equipment. Therefore, the development of simple, inexpensive, reliable and rapid methods for measuring Pb^2+^ with high sensitivity and selectivity is highly desirable.

In this respect, colorimetric methods based on functionalized gold nanoparticles (Au-NPs) are convenient, attractive, and also can satisfactorily meet these requirements. Au-NPs exhibit high extinction coefficients, strongly distance-dependent optical properties, and colors arising from Au-NPs at nanomolar concentrations allow them to be easily monitored by the naked eye without the aid of any advanced instruments. A number of Au-NP-based assays have been developed for virus [18–20], nucleic acids [21], protein [22], glucose [23], melamine [24,25], TNT [26], and heavy metal ions such as Hg^2+^, Cu^2+^, Cr^3+^ [27–29].

Recently, Au-NPs have also shown their power in the detection of lead ions [30–36]. Liu *et al*. have reported a series of pioneering experiments utilizing DNAzyme to detect Pb^2+^ in water [30,36]. The so-called DNAzyme underwent a self-cleavage process when exposed to Pb^2+^, thus inducing Au-NPs agglomeration, which could be monitored by the naked eye. A label-free DNAzyme-based sensor for Pb^2+^ detection using unmodified Au-NPs was reported [31,33]. Double-stranded DNA was not able to stabilize unmodified gold nanoparticles in the presence high concentrations of salt. With the addition of Pb^2+^, double-stranded DNA was cleaved into single-stranded fragments, which could be absorbed onto gold nanoparticles and thus prevent aggregation. The Pb^2+^ detection could be realized by the color change of the Au-NPs. Guan *et al.* investigated the pH-dependent response of citrate capped Au-NPs to Pb^2+^ ion, indicating the citrate capped Au-NPs are sensitive to Pb^2+^ ion under the pH of 11.2 [32]. Li *et al*. coated the surface of Au-NPs with a cysteine-alanine-leacine-asparagine-asparagine (CALNN) pentapeptide [33], and Pb^2+^ could be recognized by the C-terminal of the oligopeptide, thus leading to aggregation of Au-NPs. This made the detection of Pb^2+^ visible by the color change of the solution. However, the preparation of Au-NPs using citrate required heating, which is complicated and time consuming. A much milder and faster method was proposed by Yoosaf *et al*. [37], wherein gallic acid was employed to simultaneously reduce HAuCl_4_ and stabilize as-prepared Au-NPs. Then addition of Pb^2+^ caused a visual color change through “crosslinking” of Au-NPs based on Pb-GA complexation. However, the detection limit was relatively high, 5 μM, far beyond the requirements of practical applications. Tseng *et al*. further developed this method to lower the limit of detection [38]. They discovered that a narrower size distribution and minimized particle repulsion that could help to enhance Pb^2+^ detection sensitivity could be achieved by tuning the pH value of precursor solution and adding NaClO_4_ salt as destabilizer, respectively.

However, in all these experiments researchers have to make Au-NPs first, and then modify the Au-NPs or change the environment, both adding to the operational complexity. Therefore, we propose a novel way to realize lead ion detection: adding Pb^2+^ during Au-NPs synthesis. There are three steps in gold nanoparticle formation: nucleation, growth and saturation [37,39–41]. Initially, Au^3+^ is reduced to Au^0^ by gallic acid [42]. Small nuclei are formed which later aggregate into gold nanoclusters (gold seeds). During this process, gallic acid acts not only as a reducing agent, but also a stabilizer surrounding the surface of gold nanoparticles. In the growth stage, the newly-formed small gold nanoclusters are unstable and tend to agglomerate easily upon the interaction with Pb^2+^, via Pb-GA complexation. Based on this strategy, even the presence of trace Pb^2+^ results in a visual color change, therefore facilitating the colorimetric detection of trace Pb^2+^. With increasing Pb^2+^ concentration, interparticle aggregation increases, and the color would change from red to purple, and finally blue. To the best of our knowledge, this is the first report of the use of this method, that is to say adding Pb^2+^ during Au-NPs synthesis, to realize Pb^2+^ detection. Previous methods for the preparation of Au-NPs and detection of Pb^2+^ are time-consuming or expensive; while our method combines the Au-NPs synthesis and Pb^2+^ detection into one step, the Pb^2+^ detection time is greatly shortened at ambient temperature. The proposed method shows great potential as a fast, simple, and economic Pb^2+^ colorimetric sensor.

## 2. Experimental Section

### 2.1. Chemicals

Gallic acid was obtained from J&K Chemical Ltd. (Beijing, China). HAuCl_4_·4H_2_O was purchased from Sinopharm Chemical Reagent Co., Ltd. (Shanghai, China). Pb(NO_3_)_2_ and other metal ions were bought from Beijing Chemical Company (Beijing, China). The pH of the aqueous solution was adjusted to 4.5 with HCl. All reagents were of analytical grade and prepared using high pure water with a resistivity of 18 MΩ·cm.

### 2.2. Apparatus

The UV-vis spectra and kinetics were recorded on a UV-2550 spectrophotometer (Shimadzu, Japan), using 1-cm path length quartz cuvettes for measurements. The pH of the solution was measured with a PB-10 pH meter (Sartorius, Germany). The fluorescence measurement was carried out at room temperature using an LS55 fluorescence spectrometer (Perkin Elmer, USA). Transmission electron microscopy (TEM) measurements were performed with an H-7500 (Hitachi, Japan) at 80 kV. The particle size was determined by dynamic light scattering (DLS) measurements (Nano ZS). Flame atomic absorption spectroscopy (FAAS) experiment was implemented by using an AA-6800 spectrometer (Shimadzu, Japan).

### 2.3. Methods

Au-NPs were prepared by reducing HAuCl_4_ with gallic adic [37]. In contrast to previous reports, Pb^2+^ was added during the synthesis of Au-NPs. That is to say, Pb^2+^ was introduced into the system before HAuCl_4_ reacts with gallic acid. Briefly, 30 μL Pb^2+^ solutions of different concentration, 24 μL gallic acid (5.0 × 10^−2^ M), 30 μL HAuCl_4_ (2.7 × 10^−2^ M) were added in sequence to 2,916 μL deionized water (adjusted to pH 4.5 with HCl) and shaked gently for a few seconds. The solution started to change color within 20 seconds (Figure S1, Supplementary Information). Results were recorded by photographs and UV-Vis spectrophotometry. TEM samples were prepared by dropping Au-NPs solution on a carbon-coated copper grid and drying at ambient temperature. Common univalent and divalent metal ions were chosen to investigate their interference in Pb^2+^ detection, and the concentrations of metal ions studied were 5.0 × 10^−8^ M, 1.0 × 10^−7^ M, 5.0 × 10^−7^ M, and 1.0 × 10^−6^ M.

The applicability of our method in the detection of Pb^2+^ in drinking water was verified. Water samples were from our own laboratory and no pretreatment was made. We spiked the samples with standard solution containing 1.0 × 10^−5^ M to 1.0 × 10^−4^ M Pb^2+^. FAAS was then conducted to calculate recovery rate. To solve the discrepancy between the detection limits of traditional FAAS and our method, each sample was diluted 100-fold and the recovery rate recalculated using the proposed method.

## 3. Results and Discussion

### 3.1. Effect of pH

According to previous reports, the first ionization constant of gallic acid is 4.2 [43]. When pH < 4.2, electrostatic repulsion between gallic acid coated Au-NPs decreases, and the whole system is unstable. When the pH is adjusted to 4.5–5.0, gallic acid exists as monoanion, which adds to the interparticle electrostatic interaction, thus endowing the nanoparticles with good stability, so pH 4.5–5.0 is suitable for synthesizing Au-NPs. Meanwhile, Pb^2+^, compared to other metal ions, coordinates more favorably with gallic acid at pH 4.5 [37,38]. For this reason, we chose pH = 4.5 to conduct our subsequent studies.

### 3.2. Effect of Gallic Acid Concentration

**Figure 1.**
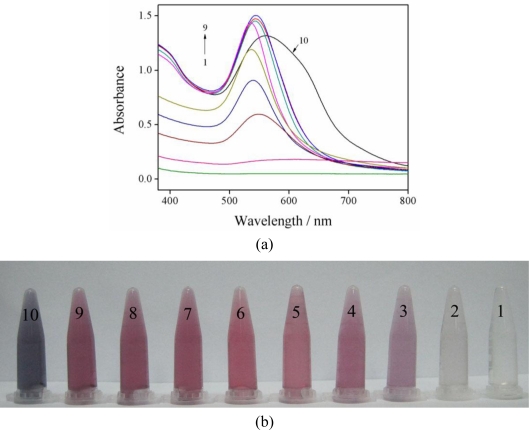
UV-vis absorption spectra **(a)** and photo images of visual color change **(b)** of gold nanoparticles prepared in the presence of different concentrations of gallic acid (1–10: 1.0 × 10^−5^ M, 2.5 × 10^−5^ M, 5.0 × 10^−5^ M, 7.5 × 10^−5^ M, 1.0 × 10^−4^ M, 2.5 × 10^−4^ M, 4.0 × 10^−4^ M, 6.0 × 10^−4^ M, 8.0 × 10^−4^ M, 1.0 × 10^−3^ M).

The effect of the concentration of gallic acid on the preparation of Au-NPs was investigated. The Au-NPs formed using different concentrations of gallic acid were characterized by UV-vis spectrophotometry. As shown in Figure 1(a), the absorbance and the position of the plasmon band of the formed Au-NPs vary with the concentration of gallic acid. When the concentration of gallic acid was low (below 2.5 × 10^−5^ M), the solution possessed a broad absorption at around 550 nm. As the gallic acid concentration increased from 5.0 × 10^−5^ M to 8.0 × 10^−4^ M, the absorption band intensified and sharpened at c.a. 540 nm. When the concentration of gallic acid is higher (1.0 × 10^−3^ M), it is clearly observed that the absorption band became broad and further red-shifted, due to the hydrogen bonding between the interparticles [37]. As shown in Figure 1(b), an obvious color change of the solution is observed. A well dispersed Au-NPs solution with red-wine color could be made from 4.0 × 10^−4^ M gallic acid, and a little mulberry color emerged at 6.0 × 10^−4^ M and 8.0 × 10^−4^ M gallic acid. In the subsequent Pb^2+^ detection, there was an obvious color change of the formed Au-NPs mixed with 5.0 × 10^−7^ M Pb^2+^ when the concentration of gallic acid was 4.0 × 10^−4^ M, while 6.0 × 10^−4^ M or 8.0 × 10^−4^ M gallic acid could not induce such a visible change. Therefore, 4.0 × 10^−4^ M gallic acid was adopted in our subsequent assays. Then DLS was used for measuring the size of as-prepared Au-NPs in the solution. The average hydrodynamic diameter of the Au-NPs capped with gallic acid was determined to be 35 nm.

### 3.3. The Colorimetric Detection of Pb^2+^

We monitored Pb^2+^ using a dual strategy of colorimetry and spectrometry. Provided that Pb^2+^ is introduced into the system before HAuCl_4_ is reduced by gallic acid, a Pb-GA complex would be formed by coordination between Pb^2+^ and the carboxylic acid group of the gallic acid [43]. Interaction between Pb^2+^ and gallic acid was confirmed by the fluorescence spectra (Figure S2, Supplementary Information), in which the addition of Pb^2+^ induced a decline of the emission at 350 nm.

**Figure 2.**
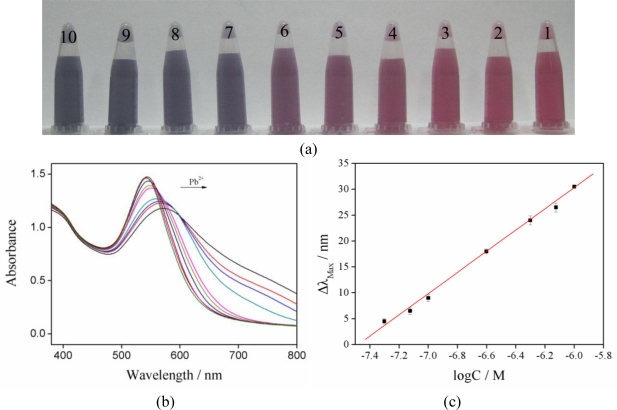
**(a)** Visual color change of Au-NPs generated upon addition of different Pb^2+^ concentrations at pH 4.5 (1–10: 0 M, 1.0 × 10^−8^ M, 2.5 × 10^−8^ M, 5.0 × 10^−8^ M, 7.5 × 10^−8^ M, 1.0 × 10^−7^ M, 2.5 × 10^−7^ M, 5.0 × 10^−7^ M, 7.5 × 10^−7^M, 1.0 × 10^−6^ M). **(b)** UV-vis absorption spectra changes of Au-NPs in the presence of Pb^2+^ concentrations. **(c)** A plot of the shift of the absorption band (Δλ) *versus* the logarithm of Pb^2+^ concentrations.

Addition of different amounts of Pb^2+^ to the system would result in a significant change of color (Figure 2(a)), from a red-wine color to purple, and finally to blue. This can be easily judged by the naked eye, even when the concentration is as low as 5.0 × 10^−8^ M. The color of the solution would perdure for a long time (Figure S3, Supplementary Information), and the difference of the colors could still be distinguished two weeks later. Quantification was realized via UV-Vis spectroscopy. From Figure 2(b), it is observed that the surface plasmon resonance (SPR) band of gallic acid capped Au-NPs without Pb^2+^ was intense at 541 nm, and the increase of Pb^2+^ concentration would induce a decrease and red shift of Au-NPs’ maximal absorption band. Figure S4 (Supplementary Information) shows the absorbance ratios (A_600_/A_541_) plot of Au-NPs against the Pb^2+^ concentration ranging from 0 to 1.0 × 10^−6^ M. The increased dotted line illustrates that Pb^2+^ can sensitively induce Au-NPs aggregation. The bathochromic shift in the plasmon resonance band (Δλ) induced by aggregation has been utilized to determine the Pb^2+^ detection limit. A good linear correlation between Δλ and logarithm of Pb^2+^ concentration was obtained in the range from 5.0 × 10^−8^ to 1.0 × 10^−6^ M, with a linear fit coefficient of 0.998, making it suitable for the quantitative determination of Pb^2+^ in aqueous solutions (Figure 2(c)). The linear equation was as follows: Δλ = 153.30018 + 20.49104 log C. The detection limit obtained in this method is 2.5 × 10^−8^ M. A concentration of Pb^2+^ of 5.0 × 10^−7^ M was chosen to conduct a reproducibility study. The relative standard deviation (RSD) was 0.44% for ten independent experiments, indicating the good reproducibility of our method.

**Figure 3.**
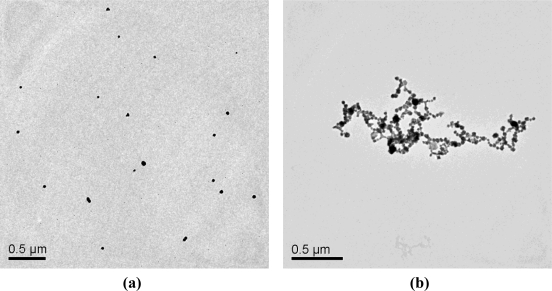
TEM images of the Au-NPs formed in the absence **(a)** and presence of **(b)** 5.0 × 10^−7^ M Pb^2+^.

Then transmission electron microscopy (TEM) of the generated Au-NPs was performed. The features of the resulting Au-NPs were directly observed from the TEM images. Figure 3 shows the TEM images of the Au-NPs in the absence and presence of 5.0 × 10^−7^ M Pb^2+^. In the absence of Pb^2+^ the Au-NPs were dispersed (Figure 3(a)), whereas the Au-NPs obviously aggregated in the presence of 5.0 × 10^−7^ M Pb^2+^ (Figure 3(b)). These results clearly indicate that the addition of trace Pb^2+^ could readily lead to aggregation of Au-NPs.

### 3.4. The Selectivity for Pb^2+^ Detection

**Figure 4.**
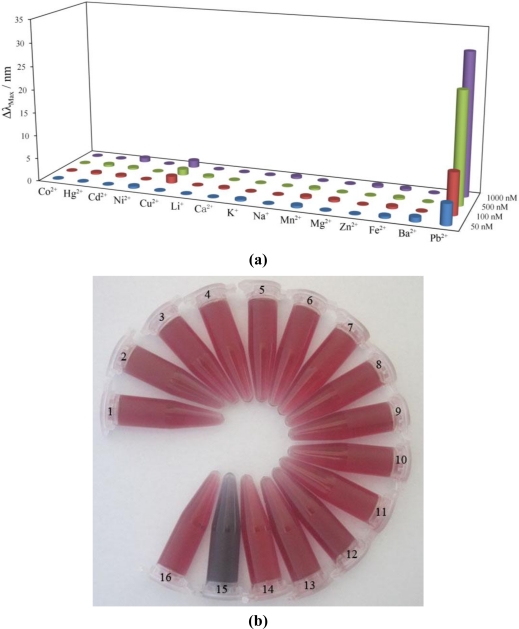
**(a)** The absorption band shift (Δλ) of the Au-NPs in the presence of Pb^2+^ or other metal ions at pH 4.5 (the concentrations of the metal ions were 5.0 × 10^−8^ M, 1.0 × 10^−7^ M, 5.0 × 10^−7^ M, and 1.0 × 10^−6^ M, respectively). **(b)** The color of the Au-NPs in the presence or absence of 5.0 × 10^−7^ M metal ions (1–16: Co^2+^, Hg^2+^, Cd^2+^, Ni^2+^, Cu^2+^, Li^+^, Ca^2+^, K^+^, Na^+^, Mn^2+^, Mg^2+^, Zn^2+^, Fe^2+^, Ba^2+^, Pb^2+^, control).

In further experiments, the selectivity of the proposed method was evaluated using other metallic cations (Li^+^, K^+^, Na^+^, Co^2+^, Hg^2+^, Cd^2+^, Ni^2+^, Cu^2+^, Ca^2+^, Mn^2+^, Mg^2+^, Zn^2+^, Fe^2+^, Ba^2+^). For comparison, different concentrations of these metallic cations were separately added in substitution of Pb^2+^ under identical conditions. The changes in the plasmon resonance band of the as-prepared Au-NPs within addition of metallic cations at the concentrations of 5.0 × 10^−8^ M, 1.0 × 10^−7^ M, 5.0 × 10^−7^ M, and 1.0 × 10^−6^ M were investigated. As shown in Figure 4(a), in the presence of Pb^2+^, a significant change in the plasmon resonance band was clearly observed, whereas no obvious change of the formed Au-NPs was detected when other metallic cations were added at the same concentration. According to Figure 4(b), it is obvious that the color of Au-NPs solution changed with the addition of 5.0 × 10^−7^ M Pb^2+^, while the Au-NPs solution remains the same red-wine color when the same amount of other metallic cations was introduced. This result was probably due to the fact that Pb^2+^ has a variable coordination number (up to 12) and flexible coordination geometry compared to other metallic ions [43–45]. Thus, one Pb^2+^ can coordinate with two or more gallic acids, but other metallic ions which possess lesser coordination numbers cannot interaction with many more phenolic hydroxyl groups because of their rigid coordination geometry [39]. This experimental result indicates the high selectivity of this Au-NPs-based assay for the detection of Pb^2+^ in aqueous solution.

### 3.5. Analytical Application in Drinking Water

To assess its applicability, the proposed method was used in the analysis of Pb^2+^ in drinking water. The sample was directly spiked with certain amounts of Pb^2+^ standard solution. The concentration of Pb^2+^ was calculated to be 6.0 × 10^−5^ M. The recovery rate was 101.4%, coinciding nicely with the result obtained by traditional FAAS, which is 102.9%, demonstrating the accuracy of the proposed method.

## 4. Conclusions

In this paper, the detection of Pb^2+^ is realized at room temperature during the synthesis of Au-NPs. That is to say, addition of Pb^2+^ leads to the formation of a Pb-GA complex, which can induce the aggregation of newly-formed small unstable gold nanoclusters. Compared with previous reports, the proposed method needs no complicated pretreatment work, such as preparation of Au-NPs of a proper size or modification of Au-NPs with certain functional groups. In particular, 5.0 × 10^−8^ M Pb^2+^ can be detected by the naked eye. A good linear correlation between the shift of the absorption band (Δλ) and logarithm of Pb^2+^ concentration was obtained in the range from 5.0 × 10^−8^ to 1.0 × 10^−6^ M, with a linear fit coefficient of 0.998. This simple, fast, and efficient method offers great potential in future onsite monitoring of Pb^2+^.

## Supplemental Information
