# Supplementary material for: Colorimetric Assay for Determination of Lead (II) Based on Its Incorporation into Gold Nanoparticles during Their Synthesis
Source: Sensors (Basel). 2010 Dec 7;10(12):11144–55. doi: 10.3390/s101211144 (PMC3231069; doi:10.3390/s101211144)

Supplemental Information

Figure S1. Kinetics of the absorbance maxima in aqueous solution of the formed Au-NPs in the presence of 5.0 × 10^{-7} M Pb^{2+}.

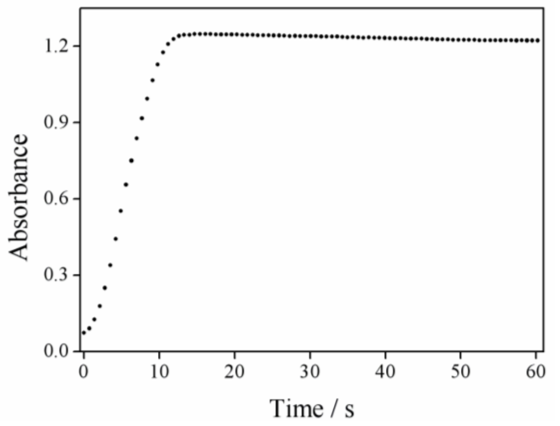

Figure S2. Fluorescence emission spectra of gallic acid (1.0 × 10^{-4} M, pH 4.5) interacted with different concentration Pb^{2+} (0 M, 1.0 × 10^{-5} M, 5.0 × 10^{-5} M, 1.0 × 10^{-4} M, 5.0 × 10^{-4} M, 1.0 × 10^{-3} M). λ_{ex} = 212 nm. Excitation and emission slit widths were set at 5.0 nm and 10.0 nm, respectively.

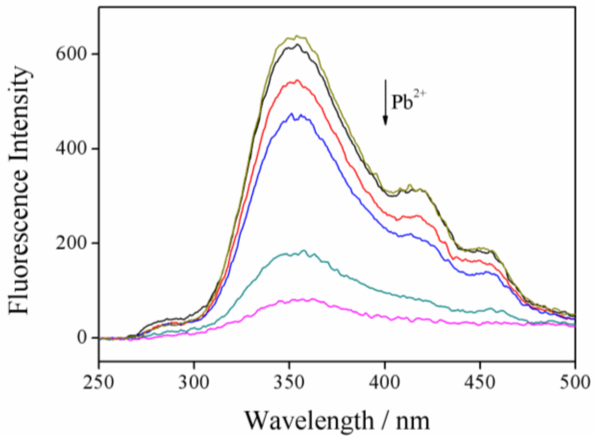

Figure S3. Plots of the relationship between the shift of the absorption band ($\Delta\lambda_{Max}$) and time over 4 h at different concentrations of Pb^{2+} (1, 1.0 × 10^{-6} M; 2, 5.0 × 10^{-7} M; 3, 1.0 × 10^{-7} M; 4, 5.0 × 10^{-8} M).

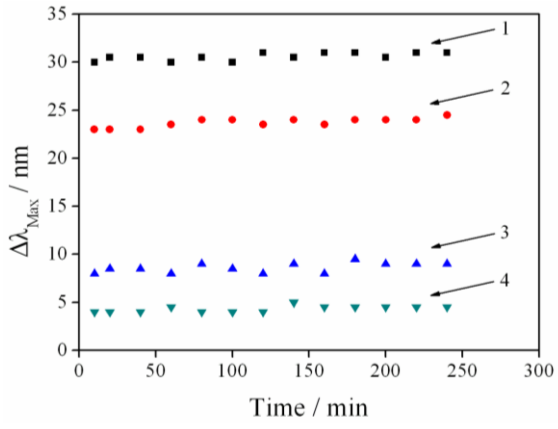

Figure S4. A plot of A_{600}/A_{541} ratios against the concentration of Pb^{2+} in the range of 0–1.0 $\times 10^{-6}$ M.

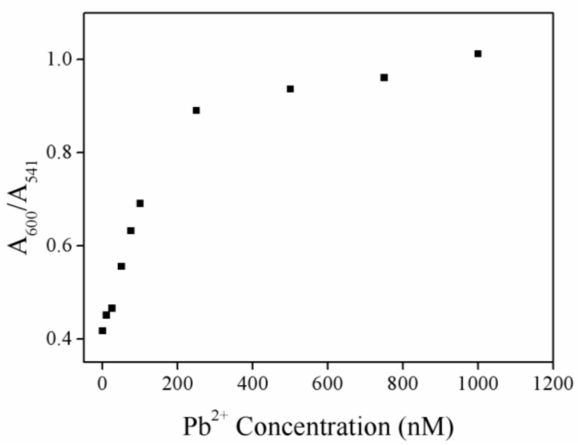

Supplement: Supplementary file 1 [file sensors-10-11144-s001.pdf]
